# First records of three *Nematocarcinus* species (Crustacea, Decapoda, Nematocarcinidae) in the deep-waters of the north-western Pacific

**DOI:** 10.3897/BDJ.10.e95101

**Published:** 2022-11-07

**Authors:** Zhibin Gan, Xinzheng Li

**Affiliations:** 1 Institute of Oceanology, Chinese Academy of Sciences, Qingdao, China Institute of Oceanology, Chinese Academy of Sciences Qingdao China; 2 Center for Ocean Mega-Science, Chinese Academy of Sciences, Qingdao, China Center for Ocean Mega-Science, Chinese Academy of Sciences Qingdao China; 3 University of Chinese Academy of Sciences, Beijing, China University of Chinese Academy of Sciences Beijing China; 4 Laboratory for Marine Biology and Biotechnology, Pilot National Laboratory for Marine Science and Technology (Qingdao), Qingdao, China Laboratory for Marine Biology and Biotechnology, Pilot National Laboratory for Marine Science and Technology (Qingdao) Qingdao China

**Keywords:** *Nematocarcinus*, new records, deep sea, the South China Sea, Kyushu-Palau Ridge

## Abstract

**Background:**

During two scientific expeditions in the South China Sea and the Kyushu-Palau Ridge area, several specimens of thread-leg shrimp were collected from deep waters. Amongst them, three species, *Nematocarcinusevansi* Burukovsky, 2000, *N.exilis* (Spence Bate, 1888) and *N.machaerophorus* Burukovsky, 2003 were newly recorded from the north-western Pacific. The morphological features of these specimens are in concordance with the original description.

**New information:**

*Nematocarcinusevansi* and *N.machaerophorus* were recorded for the second time since their original descriptions and newly found from the South China Sea. *Nematocarcinusexilis*, collected from the Kyushu-Palau Ridge area, represents a great distribution expansion from the eastern Atlantic and the Mediterranean to the Pacific, making it the fourth Atlantic-Pacific distributed *Nematocarcinus* species. Their detailed morphological characteristics, colour patterns and partial sequences of the COI and 16S rRNA genes are provided, respectively.

## Introduction

*Nematocarcinus* Milne Edwards, 1881 is the most species-rich genus amongst the caridean shrimp family Nematocarcinidae Smith, 1884, exclusively inhabiting the seafloor in deep-sea (De Grave and Fransen 2011, Burukovsky 2012, Cardoso and Burukovsky 2014). Burukovsky (2012) and Hernández-Payán and Hendrickx (2016) listed and represented 47 *Nematocarcinus* species (including 44 valid and three unofficial species). Amongst these, more than 34 species occur in the Pacific, of which about 19 species have been recorded from the north-western Pacific: *Nematocarcinusbatei* Burukovsky, 2000; *N.bituberculatus* Chace, 1986; *N.chacei* Burukovsky, 2002; *N.challengeri* Burukovsky, 2006; *N.combensis* Burukovsky, 2000; *N.crosnieri* Burukovsky, 2000; *N.gracilis* Spence Bate, 1888; *N.kaiensis* Burukovsky, 2000; *N.longirostris* Spence Bate, 1888; *N.manningi* Burukovsky, 2003; *N.nudirostris* Burukovsky, 1991; *N.parvus* Burukovsky, 2000; *N.productus* Spence Bate, 1888; *N.richeri* Burukovsky, 2000; *N.subtegulisfactus* Burukovsky, 2000; *N.subtilis* Burukovsky, 2000; *N.tenuipes* Spence Bate, 1888; *N.tenuirostris* Spence Bate, 1888; and *N.undulatipes* Spence Bate, 1888 (Spence-Bate 1888, Chace 1986, Burukovsky 2000a, Burukovsky 2000b, Burukovsky 2000c, Burukovsky 2000d, Burukovsky 2002a, Burukovsky 2003, Burukovsky 2006a, Burukovsky 2007, Burukovsky 2012).

We collected several specimens of *Nematocarcinus* during two scientific expeditions in the north-western Pacific Ocean. Morphological identification and DNA barcoding indicated that they belonged to four species: *Nematocarcinusevansi* Burukovsky, 2000, *N.exilis* (Spence Bate 1888), *N.machaerophorus* Burukovsky, 2003 and *N.undulatipes* Spence Bate, 1888. Amongst these, *N.machaerophorus* had only been recorded in the waters of the islands of Eiao and Ua Pou (Marquesas Islands, central Pacific Ocean) at depths of 1000–1100 m; *N.evansi* had only been recorded from the waters of south-western Australia at depths from 913–916 m, the Indian Ocean (Burukovsky 2003, Burukovsky 2012); both species were newly found from the South China Sea. *N.exilis* had been found in the Atlantic and the Mediterranean Sea (Crosnier and Forest 1973, Burukovsky 2012) and now have been collected from the Kyushu-Palau Ridge area. Hence, *N.evansi* and *N.exilis* were recorded from the Pacific for the first time.

We illustrated morphological characters of three *Nematocarcinus* species previously unrecorded from the north-western Pacific that were collected from the South China Sea and the Kyushu-Palau Ridge area. We included the morphological discussions for each species in the Remarks sections. Their gene sequences, colour patterns and distributions were also provided.

## Materials and methods

Specimens of *Nematocarcinusexilis* were captured during the cruise DY59 along the Kyushu-Palau Ridge area by the R/V “DA YANG HAO” in July–August 2020, captured by the ROV “Hailong IVE”. The specimens were preserved in 75% ethanol and deposited at the Sample Repository of the Second Institute of Oceanography (SRSIO), Ministry of Natural Resources of the People’s Republic of China, Hangzhou. Specimens of *N.evansi* and *N.machaerophorus* were collected during a cruise within the South China Sea by the R/V “Tan Kah Kee” in June 2020 using a deep-sea Agassiz trawl. The specimens were preserved in 75% ethanol and deposited at the Marine Biological Museum of the Chinese Academy of Sciences (MBM), Qingdao, China.

The morphological details were examined and illustrated using a stereomicroscope (Nikon SMZ1500, Japan). Total genomic DNA of the specimens was extracted from the fifth pleopod using a TIANamp Marine Animals DNA Kit (TIANGEN, China) according to the manufacturer’s instructions. Partial sequences of COI and 16S rRNA genes were amplified using polymerase chain reaction (PCR) with the primers LCO1490/HCO2198 and 16S-AR/16S-1472, respectively (Folmer et al. 1994, Crandall and Fitzpatrick 1996) and then sequenced using the same primers with an ABI 3730xl Analyzer (Applied Biosystems, Shanghai, China). The Basic Local Alignment Search Tool (BLAST, https://blast.ncbi.nlm.nih.gov/Blast.cgi) was used to detect the similarity of these sequences to the nucleotide sequence collection database in GenBank (https://www.ncbi.nlm.nih.gov). Genetic distances were calculated using the Kimura 2-parameter model in MEGA X (Kumar et al. 2018). All the sequences were deposited in GenBank.

The size of the specimens (postorbital carapace length) was measured to the nearest 0.1 mm with a vernier caliper. The following abbreviations were used: St., sampling station; CL, postorbital carapace length; AT, Agassiz trawl; Coll., collector.

## Taxon treatments

### Nematocarcinus evansi

Burukovsky, 2000

0F10EE96-A529-5E40-9109-F52D13206252

https://www.marinespecies.org/aphia.php?p=taxdetails&id=514308

Nematocarcinus evansi : Burukovsky (2000e), p. 1291, fig. 2; Burukovsky (2003), p. 85–87, fig. 23; Burukovsky (2012), p. 107–108, fig. 34.

#### Materials

- **Type status:** Other material. **Occurrence:** individualCount: 1; sex: female; lifeStage: adult (CL 29.5 mm); reproductiveCondition: non-reproductive; establishmentMeans: wild; preparations: damaged animal (ETOH), DNA extract; disposition: in collection; associatedSequences: OP093562, OP093563; occurrenceID: C836B536-48AB-5983-9712-5D897E11FE32; **Taxon:** scientificNameID: urn:lsid:marinespecies.org:taxname:514308; scientificName: *Nematocarcinusevansi* Burukovsky, 2000; order: Decapoda; family: Nematocarcinidae; genus: Nematocarcinus; specificEpithet: *evansi*; taxonRank: species; scientificNameAuthorship: Burukovsky, 2000; nomenclaturalCode: ICZN; taxonomicStatus: accepted; **Location:** higherGeography: northwestern Pacific; waterBody: Pacific Ocean; country: China; countryCode: China/CN; locality: the South China Sea; verbatimDepth: 734-736 m; verbatimCoordinates: 15.72057°N, 110.77073°E; **Identification:** identifiedBy: Zhibin Gan; dateIdentified: 12/12/2021; identificationReferences: Burukovsky 2000e, 2003, 2012; identificationRemarks: another specimen (MBM189205, female, CL 17.5 mm), *Nematocarcinusundulatipes* Spence Bate, 1888, was collected at the same sampling station and compared with *Nematocarcinusevansi* Burukovsky, 2000; **Event:** samplingProtocol: Agassiz trawl; eventDate: 13/06/2020; fieldNumber: St. AT-S58; fieldNotes: Coll. Xu; **Record Level:** language: en; collectionID: MBM189203; institutionCode: Marine Biological Museum of the Chinese Academy of Sciences (MBM)

#### Description

**Figure 1.**
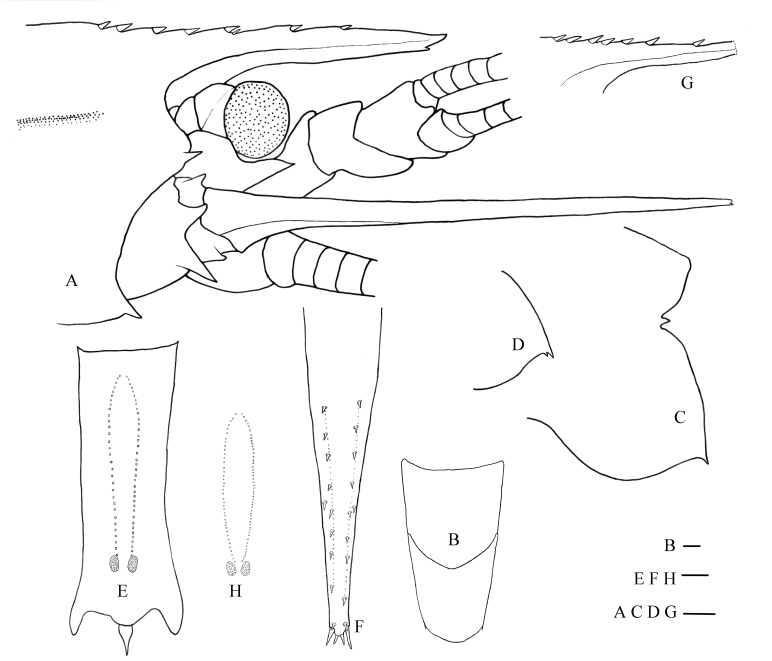
*Nematocarcinusevansi* Burukovsky, 2000, MBM189203, female (**A–F)** *N.undulatipes* Spence Bate, 1888, MBM189205, female (**G–H)**. **A** anterior part of carapace and cephalic appendages, lateral view; **B** pleomere 3–4, dorsal view; **C** left pleura of fifth pleomere, lateral view; **D** right pleura of fifth pleomere, lateral view; **E** sixth abdominal somite, ventral view; **F** telson, dorsal view; **G** rostrum, lateral view; **H** distoventral organ, ventral view. Scales: 1.0 mm.

**Figure 2.**
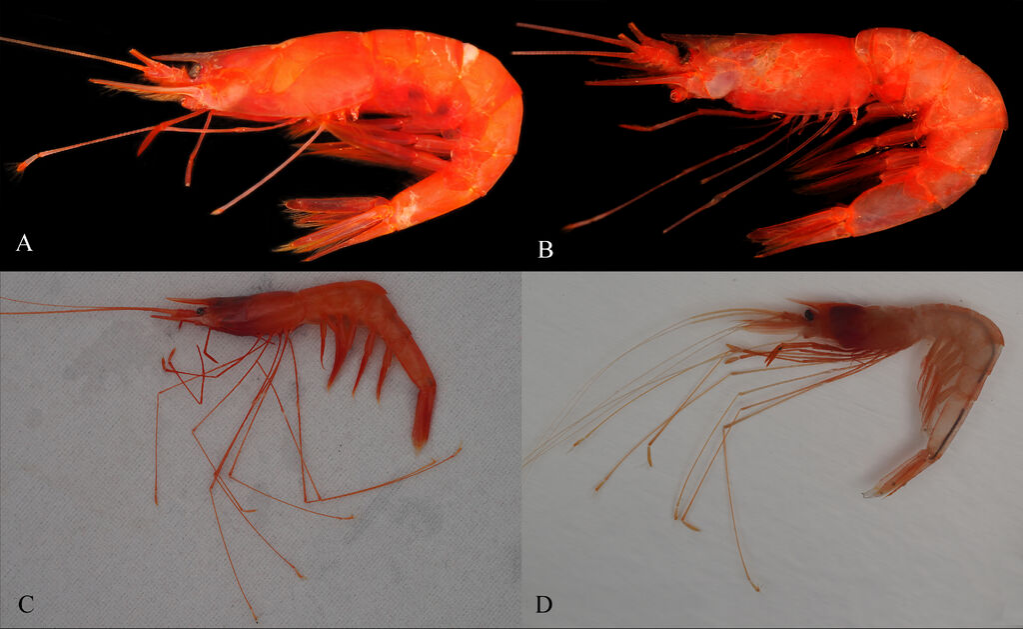
Photographs: **A** *Nematocarcinusevansi* Burukovsky, 2000, MBM189203, female; **B** *Nematocarcinusundulatipes* Spence Bate, 1888, MBM189205, female; **C** *Nematocarcinusexilis* (Spence Bate, 1888), SRSIO20080316, female; **D** *Nematocarcinusexilis* (Spence Bate, 1888), SRSIO20080316, male.

Body robust, integument hard, surface smooth, shiny. Rostrum straight, slightly over-reaching distal end of antennular peduncle, reaching mid-length of scaphocerite; 0.34 times as long as carapace (Fig. 1A and Fig. 2A); dorsal margin armed with seven articulated teeth (an additional small tooth under proximal fifth tooth), including four on rostrum proper and three on carapace posterior to orbital margin; space between teeth increasing distally, distance from apex to distal tooth longest, about one-third of its length; ventral margin armed with one small subdistal tooth (Fig. 1A). Antennal and pterygostomian teeth well developed.

Eyes normally developed; cornea wider than eye stalk.

Third maxilliped not reaching distal end of scaphocerite; ultimate segment 0.71 times as long as penultimate segment, not markedly broadened at middle, armed with four apical spinules; antepenultimate segment shorter than distal two segments combined, armed with 4–6 spines on lateral margin and two distal spines; exopod slightly shorter than antepenultimate segment.

First pereiopod slender, over-reaching the end of scaphocerite by distal half-length of carpus. Ischium of first pereiopod with four ventrolateral spines, merus unarmed; ischium of third pereiopod with one distolateral spine. Other pereiopods lost.

Posterodorsal margin of third pleomere rounded, continuation of its sides forming an angle of about 120° (Fig. 1B). Pleura of fifth pleomere without bump on inner sides, terminating by a sharp tooth curved downwards in left side (Fig. 1C), a small spine present under distal tooth on the right side (Fig. 1D).

Ventral organ at sixth pleomere formed by two single rows of long plumose setae and two spots; setae rows cambered in front half and nearly parallel in distal half, extending to front of spots; spots 1.96–2.01 times as long as wide, distance between spots about 1.04 times spots width (Fig. 1E).

Telson armed with nine pairs of dorsolateral spines, two pairs of distal spines and a pair of accessory spines in middle of telson (Fig. 1F).

Colour, bright red (Fig. 2A).

#### Distribution

Previously only known from the waters of south-western Australia (20°16'03''S, 113°13'05''E), the Indian Ocean, at depths from 913–916 m (Burukovsky 2000e, Burukovsky 2012). Presently recorded from the South China Sea, the Pacific Ocean.

#### Remarks

The present material is consistent with the original description of *N.evansi* (Burukovsky 2000e, Burukovsky 2012), especially the features of the rostrum and distoventral organ. Small differences were also observed: the South China Sea sample has a sub-spine under the proximal fifth tooth of the rostrum and distal tooth of the right pleura of the fifth pleomere. However, these should be considered individual variations. A slender specimen (MBM189205, female, CL 17.5 mm) was collected at the same sampling station, but its rostrum was broken (Fig. 1G–H Fig. 2B). It closely resembles *N.evansi* apart from its distoventral organ, which is most like that of *N.undulatipes* Spence Bate, 1888. According to Burukovsky (2012), the only difference between *N.evansi* and *N.undulatipes* is that the latter has the distance between spots of a half-spot width and the unparallel setae rows (distal part) of the distoventral organ in the sixth pleomere. The slender specimen was consistent with this and, thus, was identified as *N.undulatipes*, which was previously recorded in the South China Sea (Liu 2008). Furthermore, the COI genetic distance between these two specimens is 13.6%, only slightly less than the average genetic divergence of genus *Nematocarcinus* (16.0%, n = 20). Partial sequences of the COI and 16S rRNA genes of these two samples were deposited in GenBank for future research (OP093562, OP093563, OP089179, OP089180).

### Nematocarcinus exilis

(Spence Bate, 1888)

FD94F682-BB81-560F-AF89-870D1CB81760

https://www.marinespecies.org/aphia.php?p=taxdetails&id=107574

Stochasmus exilis : Spence-Bate (1888), p. 822–824, pl. 82, fig. 14.
Nematocarcinus exilis : Crosnier and Forest (1973), p. 116–123, fig. 32d, e; 33d, e, f; Abello and Valladares (1988), p. 100, fig. 4; Turkay (1998), p. 1787–1794, fig. 2; Burukovsky (2012), p. 108–112, fig. 35.

#### Materials

- **Type status:** Other material. **Occurrence:** individualCount: 2; sex: 1 female (DY59-I-ROV08), 1 male (DY59-I-ROV05); lifeStage: adult (female CL 21.0 mm, male CL 12.1 mm); reproductiveCondition: non-reproductive; establishmentMeans: wild; preparations: whole animal (ETOH), DNA extract; disposition: in collection; associatedSequences: OP093560, OP093561, OP089177, OP089178; occurrenceID: 6517D2A7-0EA0-5DCF-88CF-EBE8761F09E5; **Taxon:** scientificNameID: urn:lsid:marinespecies.org:taxname:107574; scientificName: *Nematocarcinusexilis* (Spence-Bate, 1888); order: Decapoda; family: Nematocarcinidae; genus: Nematocarcinus; specificEpithet: *exilis*; scientificNameAuthorship: (Spence Bate, 1888); taxonomicStatus: accepted; **Location:** higherGeography: northwestern Pacific; waterBody: Pacific Ocean; locality: the Kyushu-Palau Ridge area; verbatimDepth: 1956 m (DY59-I-ROV08), 2666 m (DY59-I-ROV05); verbatimCoordinates: 13.330868°N, 134.548018°E(DY59-I-ROV08); 16.931303°N, 134.917863°E (DY59-I-ROV08); **Identification:** identifiedBy: Zhibin Gan; dateIdentified: 06/08/2021; identificationReferences: Crosnier and Forest, 1973; Abello and Valladares, 1988; Turkay, 1998; Burukovsky, 2012; **Event:** samplingProtocol: pipet of ROV; eventDate: 20/07/2020, 02/08/2020; fieldNumber: St. DY59-I-ROV05, St. DY59-I-ROV08; fieldNotes: Coll. Gan; **Record Level:** language: en; collectionID: SRSIO20080316; institutionCode: Sample Repository of the Second Institute of Oceanography (SRSIO)

#### Description

**Figure 3.**
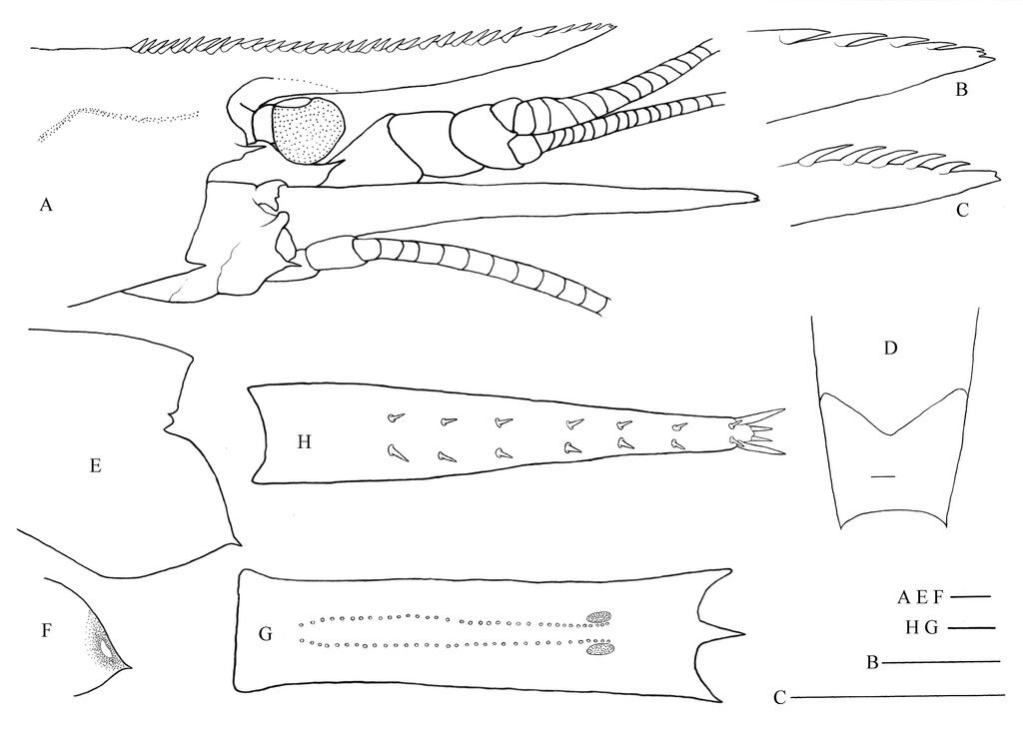
*Nematocarcinusexilis* (Spence-Bate, 1888), SRSIO20080316. **A** female, anterior part of carapace and cephalic appendages, lateral view; **B** female, distal part of rostrum, lateral view; **C** male, distal part of rostrum, lateral view; **D** female, pleomere 3–4, dorsal view; **E** female, left pleura of fifth pleomere, lateral view; **F** same, inner view; **G** female, sixth abdominal somite, ventral view; **H** female, telson, dorsal view. Scales: 1.0 mm.

Body moderately slender, integument moderately soft, surface smooth. Rostrum nearly straight, slightly upturned at tip and concave in middle. Rostrum of female specimen over-reaching distal end of antennular peduncle by one-third of its length, reaching to distal one-third of scaphocerite, about half-length of carapace (Fig. 3A and Fig. 2C); dorsal margin armed with 32 subequal teeth, including 23 on rostrum proper and nine on carapace posterior to orbital margin, distal four teeth basally sub-articulated, others all articulated; apex somewhat trifurcated (Fig. 3B); ventral margin unarmed. Rostrum of male specimen slightly over-reaching distal end of antennular peduncle, only reaching to mid-length of scaphocerite, about 0.37 times as long as carapace (Fig. 2D); dorsal margin armed with 25 subequal teeth, including 18 on rostrum proper and seven on carapace posterior to orbital margin, distal four teeth basally sub-articulated, others all articulated; apex bifid (Fig. 3C). Antennal and pterygostomian teeth well developed.

Eyes normally developed; cornea wider than eye stalk.

Third maxilliped reaching to distal quarter of scaphocerite; ultimate segment 0.76 times as long as penultimate segment, not markedly broadened at middle, armed with slender apical spinule; antepenultimate segment subequal in length to distal two segments combined, armed with 8–9 spines on lateral margin and two distal spines; exopod reaching to distal two-fifths of antepenultimate segment.

First pereiopod slender, overreaching end of scaphocerite by distal one-sixth of carpus; ischium with four ventrolateral spines, merus with 1–2 ventrolateral spines; ischium of second pereiopod with one distolateral spine, merus with 6–9 ventrolateral spines; ischium of third pereiopod with one distolateral spine, merus with 6–7 ventrolateral spines; ischium of fourth pereiopod with 0–1 distolateral spine, merus with 5–6 ventrolateral spines; ischium of fifth pereiopod unarmed, merus with 1–5 ventrolateral spines.

Posterodorsal margin of third pleomere rounded, continuation of its sides forming an angle slightly larger than 120° (Fig. 3D). Pleura of fifth pleomere with distinct bump on inner sides, terminating by a prominent tooth (Fig. 3E–F).

Ventral organ at sixth pleomere formed by two single rows of long plumose setae and two spots; setae rows nearly parallel, extending to end of spots; spots 2.47–2.61 times as long as wide, distance between spots about 2.05 times spots width (Fig. 3G).

Telson armed with seven pairs of dorsolateral spines, two pairs of distal spines, without accessory spine (Fig. 3H).

Colour, crimson red in female, faint red in male (Fig. 2C–D).

#### Distribution

Previously known in the eastern Atlantic from 62°17'N to Morocco and Canary Is-lands (900–2300 m) and the Mediterranean Sea at depths between 1033–4765 m. (Crosnier and Forest 1973, Burukovsky 2002b, Komai and Collins 2009, Burukovsky 2012). Presently recorded from the Kyushu-Palau Ridge, north-western Pacific.

#### Remarks

At present, approximately 74.5% of *Nematocarcinus* species occur in the Indo-west Pacific origin, but only three species, *N.ensifer* (SI Smith, 1882), *N.faxoni* Burukovsky, 2001 and *N.tenuipes* (also occurring in the Indian Ocean), are distributed both in the Atlantic and Pacific Oceans (Burukovsky 2012). Under these conditions, we were hesitant to identify the present specimens as *N.exilis* at first. However, the features of the present specimens, such as a relatively long rostrum extending beyond the distal end of the antennular peduncle, but falling short of the distal margin of the scaphocerite, continuation of the posterodorsal margin of the third pleomere forming an obtuse angle, pleura of the fifth pleomere with a distinct protuberance on inner sides and arrangement of the spots and setae rows of the distoventral organ, coincide with the re-descriptions of *N.exilis* by Burukovsky (2002b) and Burukovsky (2012). Komai and Collins (2009) reported a closely related species, N.sp. aff.exilis from the Manus Basin of the south-western Pacific, based on a subadult female. By comparison between the present specimens and the Manus specimen, a number of differences could be found, such as the characters of the rostrum (over-reaching distal end of antennular peduncle by one-third of its length in female, all dorsal teeth articulated or sub-articulated, rostrum apex trifurcated or bifid versus only reaching distal end of antennular peduncle in female, only posteriormost three dorsal teeth articulated, rostrum apex simple), the antepenultimate segment of the third maxilliped (8–9 lateral spines versus 12), the pleuron of the fifih pleomere (armed with prominent posteroventral tooth versus tiny posteroventral tooth), the telson (armed with seven pairs of dorsolateral spines versus five) and the most important, the distoventral organs of the sixth pleomere (setae rows extending to the distal end of spots versus only reaching the anterior ends of the spots), based on which Burukovsky (2012) considered the Manus specimen to be a novel species that differed from *N.exilis*. In view of the differences discussed above, we agree with the inference of Burukovsky (2012) that the Manus specimen may represent a species unknown to science. However, just as Komai and Collins (2009) pointed out that the Manus specimen was a subadult female, more materials are need to clarify its taxonomic status. Partial sequences of COI and 16S rRNA genes of the present specimens were deposited in GenBank for further confirmation (OP093560, OP093561, OP089177, OP089178).

### Nematocarcinus machaerophorus

Burukovsky, 2003

AB4F7160-5CD6-537C-B0EE-1A1BF2FB3426

https://www.marinespecies.org/aphia.php?p=taxdetails&id=586809

Nematocarcinus machaerophorus : Burukovsky (2003), p. 116–118, fig. 33; Burukovsky (2004), p. 1183–1184; Burukovsky (2006b), p. 1065, fig. 3a–d; Burukovsky (2012), p. 134–136, fig. 47.

#### Materials

- **Type status:** Other material. **Occurrence:** individualCount: 1; sex: female; lifeStage: adult (CL 23. 5 mm); reproductiveCondition: ovigerous; establishmentMeans: wild; preparations: damaged animal (ETOH), DNA extract; disposition: in collection; associatedSequences: OP093564, OP089181; occurrenceID: 9466BC66-651D-5D94-B040-34418CAA2A52; **Taxon:** scientificNameID: urn:lsid:marinespecies.org:taxname:586809; scientificName: *Nematocarcinusmachaerophorus* Burukovsky, 2003; order: Decapoda; family: Nematocarcinidae; genus: Nematocarcinus; specificEpithet: *machaerophorus*; scientificNameAuthorship: Burukovsky, 2003; taxonomicStatus: accepted; **Location:** higherGeography: northwestern Pacific; waterBody: Pacific Ocean; country: China; countryCode: China/CN; locality: the South China Sea; verbatimDepth: 811–849 m; verbatimCoordinates: 15.51795°N, 110.95654°E; **Identification:** identifiedBy: Zhibin Gan; dateIdentified: 06/01/2022; identificationReferences: Burukovsky, 2003, 2004, 2006b, 2012; **Event:** samplingProtocol: Agassiz trawl; eventDate: 14/06/2020; fieldNumber: St. AT-S59; fieldNotes: Coll. Xu; **Record Level:** language: en; collectionID: MBM189206; institutionCode: Marine Biological Museum ofthe Chinese Academy of Sciences (MBM)

#### Description

**Figure 4.**
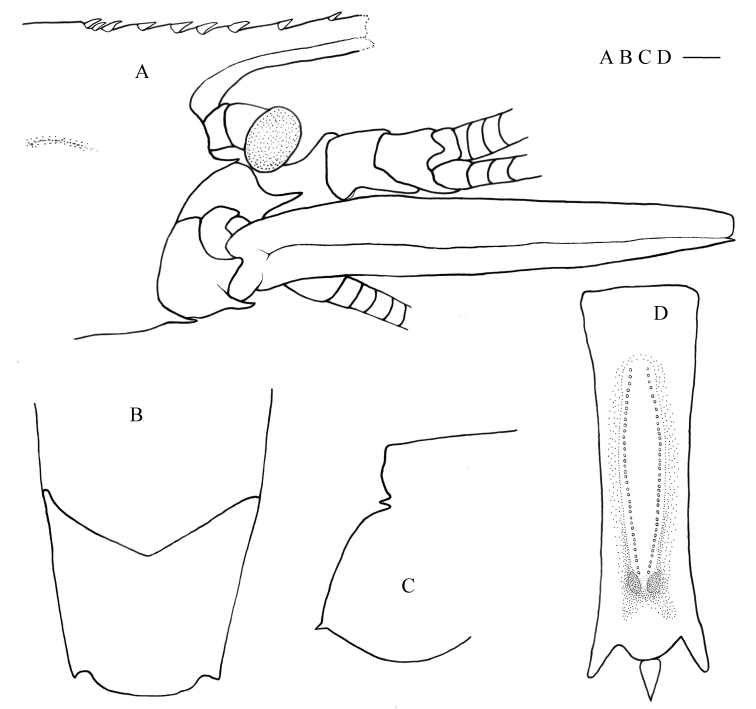
*Nematocarcinusmachaerophorus* Burukovsky, 2003, MBM189206, female. **A** anterior part of carapace and cephalic appendages, lateral view; **B** pleomere 3–4, dorsal view; **C** right pleura of fifth pleomere, lateral view; **D** sixth abdominal somite, ventral view. Scales: 1.0 mm.

Body moderately slender, integument hard, surface smooth. Rostrum damaged, remaining part nearly horizontal, dorsal margin armed with nine articulated teeth, including four on rostrum proper and five on carapace posterior to orbital margin, space between teeth increasing distally; ventral margin unarmed at remaining part (Fig. 4A). Antennal and pterygostomian teeth well developed (Fig. 4A).

Eyes normally developed; cornea wider than eye stalk.

Third maxilliped not reaching distal end of scaphocerite; ultimate segment 0.75 times as long as penultimate segment, not markedly broadened at middle, without apical spinule; antepenultimate segment subequal in length to distal two segments combined, armed with six spines on lateral margin and two distal spines; exopod falling short of distal margin of antepenultimate segment.

All pereiopods lost.

Posterodorsal margin of third pleomere rounded, continuation of its sides forming an angle slightly larger than 120° (Fig. 4B). Pleura of fifth pleomere without bump on inner sides, terminating by a sharp tooth (Fig. 4C).

Ventral organ at sixth pleomere formed by two single rows of long plumose setae and two spots; setae rows cambered laterally, extending to front of spots (Fig. 4D); spots located on a high tubercle-blister, about 1.98–2.01 times as long as wide, distance between spots slightly larger than a half of a spot width (Fig. 4D).

Telson damaged.

#### Distribution

Previously only known from type locality, the Yeiao and Ua Pou Islands (Marquesas Islands Archipelago, central Pacific) at depths of 1000–1100 m (Burukovsky 2003, Burukovsky 2004, Burukovsky 2006b, Burukovsky 2012). Presently recorded from the South China Sea, north-western Pacific.

#### Remarks

Species of *Nematocarcinus* are difficult to identify, not only because of their excessive morphological diversity, but also because of the fragility of the pereiopods and rostrum, which are usually damaged during collection. Burukovsky (2000f), Burukovsky (2000g) and Burukovsky (2012) proposed using the features of the tergum of the third pleomere, pleura of the fifth pleomere, distoventral organ of the sixth pleomere, the rostrum and the telson as key diagnostic characteristic markers. This greatly improved the taxonomy of *Nematocarcinus*. The present specimen had damaged rostrum and telson that could not be identified through morphology. Fortunately, DNA barcoding provides another efficient way to validate a species. BLAST results indicated a 98.57% match in the COI sequence between the present specimen (OP093564) and the holotype of *N.machaerophorus* Burukovsky, 2003 (KP759445, MNHN-IU-2011-5629), in which only seven nucleotide sites differed. The COI genetic distance between present specimen and the holotype of *N.machaerophorus* (KP759445, MNHN-IU-2011-5629) is 1.8%. The 16S rRNA sequence (OP089181) was identical to that of the holotype of *N.machaerophorus* (KP725571, MNHN-IU-2011-5629). Furthermore, the preserved characteristics of the present specimen, such as the tergum of the third pleomere, the pleura of the fifth pleomere and the distoventral organ, match those of *N.machaerophorus*, particularly the distoventral organ located on a high tubercle blister of the sixth pleomere. Its setae rows and spots exhibit a similar arrangement to that of the holotype (Burukovsky 2004, Burukovsky 2006b, Burukovsky 2012).

## Discussion

Shrimps of the genus *Nematocarcinus*, being obligate detritophagous and necrophagous, play an important role in the marine material energy cycle (Burukovsky 2003, Burukovsky 2012). They are commonly found from the continental slopes to the abyssal plains over 5000 m in depth (Burukovsky 1991, Burukovsky 2012, Hernández-Payán and Hendrickx 2016). However, the species identification of this group is not easy due to their varied morphological characters that sometimes overlap. It is impossible to successfully identify a specimen using the morphological method due to frequent damage of the body during collection, particularly for the long thread-like pereiopods and fragile rostrum important for identification (Hernández-Payán and Hendrickx 2016). Burukovsky (2000f), Burukovsky (2000g) and Burukovsky (2012) proposed using additional characters of the tergal angle of the third pleomere, pleural appendage of the fifth pleomere and arrangement of distoventral organ to distinguish the species, greatly facilitating the identification of *Nematocarcinus* shrimp and improving their recorded species diversity. Our report of *N.evansi* and *N.exilis* confirms these features are effective in identification. Nevertheless, some badly damaged specimens are beyond accurate morphological identification, such as the specimen of *N.machaerophorus* in the present research with its rostrum, pereiopods and telson lost during collection. It was unrecognisable based on morphology, but COI and 16S rRNA gene sequence confirmed that it belonged to the species *N.machaerophorus*. This indicates that the DNA barcoding method is efficient in taxonomy research and biodiversity monitoring. Therefore, adding DNA sequence data as one of the identifying characters for a specimen is significant.

## Supplementary Material

XML Treatment for Nematocarcinus evansi

XML Treatment for Nematocarcinus exilis

XML Treatment for Nematocarcinus machaerophorus
